# Modeling and analysis of the effect of optimal virus control on the spread of HFMD

**DOI:** 10.1038/s41598-024-56839-z

**Published:** 2024-03-16

**Authors:** Hui Wang, Weihua Li, Lei Shi, Gaofang Chen, Zhengwen Tu

**Affiliations:** 1 College of Nursing, Chongqing Three Gorges Medical College, Wanzhou, 404120 China; 2 School of Mathematics and Statistics, Guilin University of Technology, Guilin, 541004 China; 3 School of Mathematics and Statistics, Chongqing Three Gorges University, Wanzhou, 404100 China

**Keywords:** Health care, Mathematics and computing

## Abstract

A within-host and between-host hand, foot and mouth disease (HFMD) mathematical model is established and the affect of optimal control in its within-host part on HFMD transmission is studied. Through define two basic reproduction numbers, by using the fast-slow system analysis method of time scale, the global stabilities of the between-host (slow) system and within-host (fast) system are researched, respectively. An optimal control problem with drug-treatment control on coupled within-host and between-host HFMD model is formulated and analysed theoretically. Finally, the purposed optimal control measures are applied to the actual HFMD epidemic analysis in Zhejiang Province, China from April 1, 2021 to June 30, 2021. The numerical results show that the drug control strategies can reduce the virus load per capita and can effectively prevent large-scale outbreaks of HFMD.

## Introduction

HFMD is an infectious disease that tends to occur among children, which threatens children’s physical and mental health, and even causes even causes death. The main pathogenic viruses are Coxsackie virus A16 (Cox A16) and enterovirus 71 (EV 71)^1,2^. HFMD occurs mostly in Asia^3–9^. Recently, mathematical models based on compartmental structure are often used to analyse the transmission mechanism of HFMD. There were two types of dynamic mathematical models involving between and within hosts for HFMD. On the one hand, for between-host models, the authors applied SIR model to analyse the actual outbreak of HFMD in some Asian cities^2,3^. And then, SIR model in Refs.^2,3^ has been extended to many complex models that take into account more factors related to HFMD transmission, such as incubation period^10,11^, isolation measures^12,13^, viruses in contaminated environments^14–16^, seasonal factors^15,17–19^, vaccination^20,21^, asymptomatic infections^11,18,22^, stage structure^23^ and so on. On the other hand, for within-host models, based on experimental data, the authors proposed a virus dynamics model to analyze the evolution of EV71 in human leiomyoma cells^24^. Unfortunately, to the best of our knowledge, there are fewer studies on that jointly consider in-host and between-host dynamics modeling for HFMD. A natural question is: how does the within-host evolution of the pathogens impact the transmission of HFMD at the population-level? How does the evolution of pathogens in the host affect the spread of HFMD at the population level? The authors have provided a reference method to solve the above problems^25^. The optimal control strategies were introduced into coupled within-host and between-host HIV model to discuss the affect of virus load per capita on the spread of HIV at the population level^25^.

The optimal control theories have been successfully applied to HFMD mathematical models. The authors applied optimal control theories in between-host models to derive optimal control strategies with respect to treatment and quarantine controls^16,26^. It is very interesting to extend the optimal control methods of Refs.^16,26^ to study a coupled within-host and between-host HFMD model, especially optimal controls are only introduced into the within-host portion of the coupled model. Moreover, although the scholars studied the optimal control for HIV which is a different infectious diseases from HFMD^25^, it is very interesting that its optimal control research methods, in which optimal control strategies were introduced into coupled within-host and between-host HIV model, are extended to study the optimal control of HFMD. Therefore, it is possible to combine the research methods explored in Refs.^16,25,26^ and further expand it to the research of multi-scale optimal control of hand, foot and mouth disease.

This paper focuses on optimal control in multi-scale HFMD model, and its main contributions are listed as:

1. To find out the law of the impact of the within-host virus load on HFMD transmission, a novel multi-scale HFMD model involving the within-host and between-host dynamics is proposed.
2. The fast-slow analysis approach developed in Ref.^27^ is extended to study dynamic behaviors of the purposed multi-scale HFMD model. Note that for our model, at the between-host level, the combined impact of human-human and environment-human infections on the spread of HFMD, which brings new challenges to dynamics analysis.
3. The optimal control with drug-treatment control on the within-host part of the purposed multi-scale HFMD system is formulated, and analysed theoretically. In addition, the related optimal control strategies are applied to discuss the actual HFMD outbreak in Zhejiang Province, China from April 1, 2021 to June 30, 2021.

## Model formulation

This paper considers two time scales of HFMD transmission: a slow time scale with respect to between-host dynamics and a fast time scale with respect to within-host dynamics. Hence, HFMD infection involves different time scales. A multi-scale HFMD model that couples the between-host and within host dynamics will be presented.

On the slow time scale, we consider two main types of transmission: direct transmission (human to human) and indirect transmission (human to environmental virus), by referring to the modeling of HFMD in Refs.^20,21^. Besides, the modeling further introduces that for a infected individual, the transmission capacity to a susceptible individual and the rate of virus shedding to the living environment depend on the dynamic change of per capita virus load. The total population size *N*(*t*) is divided to susceptible *S*(*t*), exposed *E*(*t*), infected *I*(*t*), recovered *R*(*t*), and denote the total population size $N(t)=S(t)+E(t)+I(t)+R(t)$. Similar to Refs.^14,15^, define that *W*(*t*) is the density of pathogen of the contaminated environments including door handles, towels, handkerchiefs, toys, utensils, bedding, underclothes and so on at time *t*. Then, the between-host model is established as following:

1 $$\begin{aligned} \left\{ \begin{aligned}&\frac{dS}{dt}=\Lambda -\beta (V)SI-\sigma SW-\iota S+\eta R,\\ {}&\frac{dE}{dt}=\beta (V)SI+\sigma SW- (\varsigma +\iota )E,\\ {}&\frac{dI}{dt}=\varsigma E-(\lambda +\iota )I,\\&\frac{dR}{dt}=\lambda I-(\eta +\iota )R,\\&\frac{dW}{dt}=\gamma (V)I-\xi W,\\ \end{aligned}\quad \right. \end{aligned}$$

where $\Lambda ,\sigma ,\iota ,\varsigma ,\lambda ,\eta$ are all positive constants, and $\beta (V)$ is increasing function with *V* (*V* is the average density of enterovirus in a host). $\gamma (V)$ is the shedding rate that depends on viral load in a host. $\xi$ is the clearance rate of enterovirus in the polluted environment. All the parameter definitions are listed in Table 1.

**Table 1:** Biological meaning of parameters.

Parameter	Meaning
$\Lambda$	Recruitment rate
$\beta (V)$	Transmission rate from *I*(*t*) to *S*(*t*)
$\sigma$	Transmission rate from *W*(*t*) to *S*(*t*)
$\iota$	Natural leaving rate
$\eta$	Rate from *R*(*t*) to *S*(*t*)
$1/\varsigma$	Average incubation period
$\lambda$	Recovery rate of *I*(*t*)
$\gamma (V)$	Shedding rate that depends on viral load within a host
$\xi$	Clearance rate of pathogens *W*(*t*)
$\Lambda _T$	Recruitment rate of target cells
*d*	Cell infection mortality
$\mu$	Natural cell mortality
*p*	Rate of virus particles per infected cell
$\tilde{\beta }$	Transmission rate of cells from *V*(*t*) to *T*(*t*)
*c*	Mortality rate of enterovirus

On the fast time scale, the modeling mainly refers to the research results in Ref.^24^. Based on the experimental analysis of HFMD pathogenic virus in a cell culture environment, let *T* be the average density of target (uninfected) cells^24^, $T^{E}$ be the average density of ecliptic cells, $T^{I}$ be the average density of virus-producing cells or infectious cells, and *V* be the average density of enterovirus. In order to simplify the theoretical analysis later, similar to the modeling methods^24,25^, we do not distinguish between the types of enteroviruses. It always assume that *V* represents the average load of various viruses in infected individuals, mean while the transmission ability of infected individuals is often related to the virus load level in the human individuals. Then, the following HFMD virus model is established:

2 $$\begin{aligned} \left\{ \begin{aligned}&\frac{dT}{dt}=g(T)-\tilde{\beta } VE,\\&\frac{dT^{E}}{dt}=\tilde{\beta }VE-\zeta T^{E},\\&\frac{dT^{I}}{dt}=\zeta T^{E}-dT^{I},\\ {}&\frac{dV}{dt}=pT^{I}-cV,\\ \end{aligned}\quad \right. \end{aligned}$$

where *g*(*T*) represents the cell recruitment rate function depended on *T*, $\zeta$ represents the conversion rate from $T^{E}$ to $T^{I}$ and other parameter definitions are listed in Table 1. Note that for the system (Eq. 2), $T^{E}$ can completely convert into $T^{I}$. Hence, for simplicity, the second equation of the system (Eq. 2) can be removed through simple changes, which does not affect its dynamic behavior of the model. Consider  the evolution of cells and viruses in an individual in a short period of time, and let $g(T)=\Lambda$, where $\Lambda$ is a positive constant and assume that the natural cell mortality is a positive constant $\mu$. Then, by modifying the model (Eq. 2), the within-host dynamics of an average infected individual for HFMD are described by

3 $$\begin{aligned} \left\{ \begin{aligned}&\epsilon \frac{dT}{dt}=\Lambda _{T}-\tilde{\beta } VT-\mu T,\\&\epsilon \frac{dT^{I}}{dt}=\tilde{\beta }VT-(\mu +d)T^{I},\\&\epsilon \frac{dV}{dt}=pT^{I}-cV,\\ \end{aligned}\quad \right. \end{aligned}$$

where $\epsilon$ denotes the ratio of the fast time scale to the slow time scale, and  $0<\epsilon \ll 1$.

### Remark 1

The obvious difference between the between-host model (Eq. 1) and the model in Refs.^16,26^ is that the transmission rate $\beta (V)$ and the shedding rate $\gamma (V)$ are variable functions of the virus load per capita *V*(*t*), while those in previous studies are usually constants or only periodic functions of time *t*. Therefore, it brings new challenges to theoretical analysis and numerical experiments.

### Remark 2

The within model (Eq. 3) without time scale parameter $\epsilon$ is a very classic virus dynamic model^28^. Therefore, the dynamic behaviors of the model (Eq. 3) are very easy to be analysed based on previous studies, which is conducive to study the coupling model of Eqs. (1) and (3).

## Analysis of the disease dynamics

Because the systems (Eq. 1) and (Eq. 3) present different time scales, their dynamic behaviors would be analysed by using the fast-slow system analysis method in Ref.^27^. The coupling model of Eqs. (1) and (3) can be divided into a fast system and a slow system.

### The fast system

The system (Eq. 3) is not affected by the system (Eq. 1), so the dynamics of the system (Eq. 3) can be discussed separately. The system (Eq. 3) always has a disease-free equilibrium

$$\begin{aligned} F_{0}=(T_{0},0,0)=\left( \frac{\Lambda _{T}}{\mu },0,0 \right) . \end{aligned}$$

By using the next generation matrix method in Ref.^29^, the basic reproduction number for the system (Eq. 3) is calculated as

4 $$\begin{aligned} R_{v}=\frac{\tilde{\beta }pT_{0}}{c(\mu +d)}. \end{aligned}$$

Moreover, if $R_{v}>1$, then the system (Eq. 3) has a unique positive equilibrium

$$\begin{aligned} F_{*}=(T_{*},T_{*}^{I},V_{*})=\bigg (\frac{T_{0}}{R_{v}},\frac{c\mu (R_{v}-1)}{p\tilde{\beta }},\frac{\mu (R_{v}-1)}{\tilde{\beta }}\bigg ). \end{aligned}$$

#### Lemma 1

$\Gamma$ is a positively invariant set of the system (Eq. 3), where

$$\begin{aligned} \Gamma =&\bigg \{\big (T(t),T^{I}(t),V(t)\big )\in \mathbb {R}^{3}_{+}: 0\le T(t)+T^{I}(t)\le \frac{\Lambda _{T}}{\mu } , 0\le V(t) \le \frac{p\Lambda _{T}}{\mu c}\bigg \}. \end{aligned}$$

#### Proof

When the state variables of the system (Eq. 3) are all equal to 0, the derivative of each variable is greater than or equal to 0. Hence, we obtain that $T(t)\ge 0,T^{I}(t)\ge 0,V(t)\ge 0$. Let $N_{T}(t)=T(t)+T^{I}(t)$, we obtain $N_{T}(t)\ge 0$. From the system (Eq. 3), the total size $N_{T}(t)$ satisfies the following equation

5 $$\begin{aligned} \frac{dN_{T}}{dt}= & {} \frac{1}{\epsilon }(\Lambda _{T}-\mu T-(\mu +d)T^{I}) \nonumber \\ {}= & {} \frac{1}{\epsilon }(\Lambda -\mu N_{T}-dT^{I})\nonumber \\ {}\le & {} \frac{1}{\epsilon }(\Lambda -\mu N_{T}). \end{aligned}$$

According to the comparison theorem and let $\psi (0)$ be the initial condition of the system (Eq. 3), if $\psi (0)\in \Gamma$, then it can be obtained that $N_{T}\le \frac{\Lambda _{T}}{\mu }$.

Moreover, if $\psi (0)\in \Gamma$, then for the last equation of the system (Eq. 3), we have

6 $$\begin{aligned} \frac{dV}{dt}= & {} \frac{1}{\epsilon }(pT^{I}-cV) \le \frac{1}{\epsilon } \left( p\frac{\Lambda _{T}}{\mu }-c V \right) . \end{aligned}$$

Similarly, we have $V(t) \le \frac{p\Lambda _{T}}{\mu c}$. Therefore, $\Gamma$ is a positively invariant set of the system (Eq. 3). $\square$

#### Theorem 1

If $R_{v}<1$, then the disease-free equilibrium $F_{0}$ of the system (Eq. 3) is globally asymptotically stable.

#### Proof

Consider the following Lyapunov function

$$\begin{aligned} L(T,T^{I},V)=\epsilon T_{0} \left( \frac{T}{T_{0}}-\ln \frac{T}{T_{0}}-1 \right) +\epsilon T^{I}+\frac{\epsilon (\mu +d)}{p}V. \end{aligned}$$

Take the derivative of $L(T,T^{I},V)$ along the trajectory line of the system (Eq. 3) as

$$\begin{aligned} \frac{dL}{dt}&=\epsilon \Lambda _{T} \left( 2-\frac{T}{T_{0}}-\frac{T_{0}}{T} \right) +\frac{\epsilon c(\mu +d)}{p}(R_{v}-1)V\le 0. \end{aligned}$$

It is obvious that $\frac{dL}{dt}=0$ if and only if $\psi (t)=F_{0}$. According to the Lasalle invariance principle, one has that $F_{0}$ of the system (Eq. 3) is globally asymptotically stable as $R_{v}<1$. This completes the proof. $\square$

Next, when $\psi (0)\ne F_{0}$, we have the following result.

#### Theorem 2

Assume that $\psi (0)\ne F_{0}$ of the system (Eq. 3) holds. If $R_{v}>1$, then the positive equilibrium $F_{*}$ of the system (3) is globally asymptotically stable.

#### Proof

Define the following Lyapunov function

$$\begin{aligned} U(T,T^{I},V)=&\epsilon T_{*} \left( \frac{T}{T_{*}}-\ln \frac{T}{T_{*}}-1 \right) +\epsilon T_{*}^{*} \left( \frac{T^{I}}{T_{*}^{I}}-\ln \frac{T^{I}}{T_{*}^{I}}-1 \right) \\&+\epsilon \frac{(\mu +d)}{p}V_{*} \left( \frac{V}{V_{*}}-\ln \frac{V}{V_{*}}-1 \right) . \end{aligned}$$

*U* has the time derivative along the de-trajectory line of the system (Eq. 3) as

$$\begin{aligned} \frac{dU}{dt}&=\epsilon \mu T_{*} \left( 2-\frac{T}{T_{*}}-\frac{T_{*}}{T} \right) +\epsilon (\mu +d)T_{*}^{I} \left( 3-\frac{T_{*}}{T}-\frac{TT_{*}^{I}V}{T_{*}T^{I} V_{*}}-\frac{T^{I}V_{*}}{T_{*}^{I}V} \right) \le 0. \end{aligned}$$

$\frac{dU}{dt}=0$ if and only if $\psi (t)=F_{0}$. Similarly, we obtain that $F_{*}$ of the system (Eq. 3) is globally asymptotically stable as $R_{v}>1$. This completes the proof. $\square$

#### Remark 3

There is little difference between the system (Eq. 3) and the classical virus dynamic model in Ref.^28^. Therefore, for simplicity, the detailed derivation processes related to Lyapunov functions $L(T,T^{I},V)$ in Theorem 1 and $U(T,T^{I},V)$ in Theorem 2 are omitted, which can be refer to Ref.^28^.

### The slow system

The between-host system (Eq. 1) is on a far slower time scale than the within-host system (Eq. 3). If the state variables of the within-host system (Eq. 3) can quickly stabilize to an equilibrium state, then we can discuss the dynamics of the between-host system (Eq. 1) based on the steady state of the within-host system (Eq. 3). In fact, the average duration of an infected HFMD individual’s viral load in an unstable state is slightly less than 1 day in Ref.^24^, which is short time. Next, we only study the dynamic behaviors of the system (Eq. 1) with the case that the $R_{v}>1$ with respect to the system (Eq. 3) (For the case $R_{v}<1$, the disease can not spread in the hosts). Hence, for the system (Eq. 1), letting $\beta (V)=\beta (V_{*})$ and $\gamma (V)=\gamma (V_{*})$, where $V_{*}=\frac{m(R_{v}-1)}{k}$, if $R_{v}>1$, then from the system (Eq. 1), we have

7 $$\begin{aligned} \left\{ \begin{aligned}&\frac{dS}{dt}=\Lambda -\beta (V_{*})SI-\sigma SW-\iota S+\eta R,\\&\frac{dE}{dt}=\beta (V_{*})SI+\sigma SW- (\varsigma +\iota )E,\\&\frac{dI}{dt}=\varsigma E-(\lambda +\iota )I,\\&\frac{dR}{dt}=\lambda I-(\eta +\iota )R,\\&\frac{dW}{dt}=\gamma (V_{*})I-{\xi W.}\\ \end{aligned}\quad \right. \end{aligned}$$

Then, for between-host system (Eq. 1), it only needs to discuss dynamics of system (Eq. 7).

#### Lemma 2

*X* is a positively invariant set of the system (Eq. 7), where

$$\begin{aligned} X=&\bigg \{\big (S(t),E(t),I(t),R(t),W(t)\big )\in \mathbb {R}^{5}_{+}: 0\le S(t)+E(t)+I(t)+R(t)\le \frac{\Lambda }{\iota } , 0\le W(t) \le \frac{\Lambda \gamma (V_{*})}{\iota \xi }\bigg \}. \end{aligned}$$

#### Proof

It is easy to verify this conclusion as the similar proof process to Lemma 1. Its proof is omitted here.

It is obvious that the system (Eq. 7) has a disease-free equilibrium

$$\begin{aligned} E_{0}=(S_{0},0,0,0,0)=(\frac{\Lambda }{\iota },0,0,0,0). \end{aligned}$$

By using the calculation method in Ref.^29^, the basic reproduction number for the system (7) is

8 $$\begin{aligned} R_{h}=\frac{\varsigma \beta (V_{*})S_{0}}{(\lambda +\iota )(\varsigma +\iota )}+ \frac{\sigma \varsigma \gamma (V_{*})S_{0}}{\xi (\lambda +\iota )(\varsigma +\iota )}. \end{aligned}$$

$$\square$$

#### Theorem 3

The disease-free equilibrium $E_{0}$ of the system (Eq. 7) is globally asymptotically stable as $R_{h}<1$ and it is unstable as $R_{h}>1$.

#### Proof

According to the proof process of Theorem 2 in Ref.^29^, it is clearly that $E_{0}$ is stable (unstable) as $R_{h} < 1$ ($R_{h} >1$).

Next, we will prove that $E_{0}$ is globally attractive. According to Lemma 2, X is a positively invariant set for the system (7). For the system (Eq. 7) with the initial value $\varphi (0)\in X$, there exists any positive constant $\varepsilon _{1}>0$ such that one has that

$$\begin{aligned} S\le \frac{\Lambda }{\iota }+\varepsilon _{1}, \text {for } t\ge 0. \end{aligned}$$

Hence, for all $t\ge 0$, if $R_{h} < 1$, then it follows from the system (Eq. 7) that

9 $$\begin{aligned} \left\{ \begin{aligned}&\frac{dE}{dt}\le \beta (V_{*}) \left( \frac{\Lambda }{\iota }+\varepsilon _{1} \right) I +\sigma \left( \frac{\Lambda }{\iota }+\varepsilon _{1} \right) W- (\varsigma +\iota )E,\\&\frac{dI}{dt}\le \varsigma E-(\lambda +\iota )I,\\&\frac{dW}{dt}\le \gamma (V_{*})I-\xi W.\\ \end{aligned}\quad \right. \end{aligned}$$

Let $u=(u_{1},u_{2},u_{3})^{T}$, it can obtain the auxiliary system

$$\begin{aligned} \frac{du}{dt}=(J+\varepsilon _{1}M_{1})u, \end{aligned}$$

where

10 $$\begin{aligned} M_{1}=\left( \begin{array}{lll} 0 &{} \beta (V_{*}) &{} \sigma \\ 0&{}0&{}0 \\ 0&{}0&{}0 \end{array} \right) . \end{aligned}$$

Let *s*(*J*) be the maximum real part of all the eigenvalue for *J*. According to the proof process of Theorem 2 in Ref.^29^, if $R_{h}<1$, which means that $s(J)<0$, then $s(J)<0$ for a small enough positive constant $\varepsilon _{1}$. According to the comparison theorem, as $t\rightarrow \infty$, it follows that

$$\begin{aligned} (E,I,W)^{T}\le (u_{1},u_{2},u_{3})^{T}\rightarrow 0. \end{aligned}$$

From above equation, we also easily obtain that $R\rightarrow 0$ and $S\rightarrow S_{0}=\frac{\Lambda }{\iota }$. That is, $E_{0}$ is globally asymptotically stable as $R_{h}<1$. This completes the proof. $\square$

Next, we will prove that the system (Eq. 7) is uniformly persist. Denote

$$\begin{aligned}&X_{0}=\{(S,E,I,R,W)\in X:E>0,I>0,R>0,W>0\},\\&\partial X_{0}=X \backslash X_{0}. \end{aligned}$$

It is clear that $X_{0}$ and $\partial X_{0}$ are all positively invariant sets. Then, drawing on the relevant research works of Refs.^30,31^, we give the following conclusion.

#### Theorem 4

If $R_{h}>1$, then the system (Eq. 7) is uniformly weakly persistent. That is, if $R_{h}>1$, then there exists $\delta >0$ such that the solution $\varphi (t)$ of the system (Eq. 7) with any initial value $\varphi (0)\in X_{0}$ satisfies $\limsup \limits _{t \rightarrow +\infty }(E,I,W)>\delta.$

#### Proof

Let $G_{\partial }= \{\varphi (0)\in \partial X_{0}:\varphi (t)\in \partial X_{0},\forall t\ge 0\}.$ First, we prove that $G_{\partial }=\{(S,0,0,0,0)\in \partial X _{0} :S\ge 0\}\triangleq G'_{\partial }.$ $\square$

It is obvious that $G'_{\partial }\subseteq G_{\partial }$, and thus, it only needs to prove $G_{\partial }\subseteq G'_{\partial }$. Suppose that $G_{\partial }\not \subseteq G'_{\partial }$, then arbitrary solution $\varphi (t)$ of the system (Eq. 7) with the initial value $\varphi (0)\in \partial X_{0}$ satisfies

$$\begin{aligned} \varphi (t)\in G_{\partial } \text { and } \varphi (t)\not \in G'_{\partial }. \end{aligned}$$

From the system (Eq. 7), at least one of *E*(*t*), *I*(*t*), *R*(*t*) and *W*(*t*) is not zero. Without loss of generality, assume that $E(t)=0, I(t)=0, R(t)=0$, but $W(t)>0$. For the system (Eq. 7), it derives that for $\forall t>0$,

11 $$\begin{aligned}&E(t)=e^{-(\varsigma +\iota )t}\bigg [E(0)+\int _{0}^{t} \big [\beta (V_{*})S(u)I(u)+\sigma S(u)W(u)]du\bigg ]>0, \end{aligned}$$

12 $$\begin{aligned}&I(t)=e^{-(\varsigma +\iota )t}\big [I(0)+\int _{0}^{t}\varsigma E(u)du \big ]>0, \end{aligned}$$

13 $$\begin{aligned}&R(t)=e^{-(\eta +\iota ) t}\big [R(0)+ \int _{0}^{t}\lambda I(u)du \big ]>0, \end{aligned}$$

14 $$\begin{aligned}&W(t)=e^{-\xi t}\big [W(0)+\int _{0}^{t}\gamma (V_{*})I(u)du\big ]>0. \end{aligned}$$

Obviously, it follows from Eq. (11) that $\varphi (t) \not \in \partial X_{0},t>0$, which contradicts the hypothetical condition $\varphi (t)\in G_{\partial }$. For other cases, the similar contradictory conclusions can also be obtained. Therefore, one has that $G_{\partial }\subseteq G'_{\partial }$. Moreover, it follows from $G_{\partial }=G'_{\partial }$ that $G_{\partial }$ only has the disease-free equilibrium $E_{0}(S_{0},0,0,0,0)$, meanwhile $E_{0}$ is a compact and isolate invariant for $\varphi (0)\in G_{\partial }$.

Denote $W^{s}(E_{0})$ is the stable manifold of $E_{0}$. Next, we prove that $W^{s}(E_{0})\cap X_{0}=\emptyset$ if $R_{h}>1$. We only need to prove that there exists a constant $\varepsilon >0$ such that if $R_{h}>1$, then one has

$$\begin{aligned} D(\Phi _{t}(\varphi (0)),E_{0})^{\infty }\ge \varepsilon , \end{aligned}$$

where *D* is a distance function in $X_{0}$, $\Phi _{t}(\varphi (0))$ is an arbitrary solution of the system (Eq. 7), and $\varphi (0)\in X_{0}$. If it is assumed that the above conclusion are not true, then there must exist $\bar{\varepsilon }>0,T>0$ such that $D(\Phi _{t}(\varphi (0)),E_{0})^{\infty }<\bar{\varepsilon }$, for $t>T$. In this case, for $t>T$, one has $\frac{\Lambda }{\iota }-\bar{\varepsilon } \le S(t)\le \frac{\Lambda }{\iota }+\bar{\varepsilon }$, $0\le E(t)\le \bar{\varepsilon }$, $0\le I(t)\le \bar{\varepsilon }$, $0\le R(t)\le \bar{\varepsilon }$ and $0\le W(t)\le \bar{\varepsilon }$. Hence, for all $t\ge 0$, if $R_{h} < 1$, then by the system (Eq. 7), we have

15 $$\begin{aligned} \left\{ \begin{aligned}&\frac{dE}{dt}\ge \beta (V_{*}) \left( \frac{\Lambda }{\iota }+ \bar{\varepsilon } \right) I +\sigma \left( \frac{\Lambda }{\iota }+\bar{\varepsilon } \right) W- (\varsigma +\iota )E,\\&\frac{dI}{dt}\ge \varsigma E-(\lambda +\iota )I,\\&\frac{dW}{dt}\ge \gamma (V_{*})I-\xi W.\\ \end{aligned}\quad \right. \end{aligned}$$

Letting $\overline{u}=(\overline{u}_{1},\overline{u}_{2},\overline{u}_{3})^{T}$, we get the following auxiliary system

$$\begin{aligned} \dot{\overline{u}}=(\tilde{J}+\bar{\varepsilon }M_{1})\overline{u}. \end{aligned}$$

Denote $s(\tilde{J})$ be the maximum real part of all the eigenvalue for $\tilde{J}$. It should note that the auxiliary system $\dot{\overline{u}}=\tilde{J}\overline{u}$ satisfies all the necessary conditions of Theorem 2 in Ref.^29^. Then, according to Theorem 2 in Ref.^29^, $R_{h}>1$ yields $s(\tilde{J})<0$. Hence, there exists a small enough $\bar{\varepsilon }$ such that $s(J+\bar{\varepsilon })<0$. According to the comparison theorem, when $t\rightarrow \infty$, it follows that

$$\begin{aligned} (E,I,W)^{T}\ge (u_{1},u_{2},u_{3})^{T}\rightarrow \infty , \end{aligned}$$

which contracts with our assumption. Hence, $W^{s}(E_{0})\cap X_{0}=\emptyset$ as $R_{h}>1$. Thus, the system (Eq. 7) is uniformly weakly persistent. This completes the proof.

## Optimal control

This section will discuss optimal control of multi-scale HFMD model and the affect of optimal control in its within-host part on HFMD transmission. Unlike the previous researches [16,26], in which the optimal controls were only introduced into between-host model, we discuss the optimal control strategies in a coupled within-host and between-host model for HFMD, especially optimal controls are only introduced into the within-host part of the coupled model. In addition, the schloars have analysed the effects of six different drug treatments including reduning, tanreqing, xiyanping, yanhuning, ribavirin, and combining Bhavelin and Renzen on HFMD virus in human body^32,33^. Their research results showed that different drug treatments have different effects, and the more expensive the drug results in the better the treatment effect, i.e., the more expensive the drug is conducive to controlling the spread of the virus in the human body. Then, the coupled model by combing between-host model (Eq. 1) and within-host model (Eq. 3) is as follow:

16 $$\begin{aligned} \left\{ \begin{aligned}&\frac{dS}{dt}=\Lambda -\beta (V)SI-\sigma SW-\iota S+\eta R,\\&\frac{dE}{dt}=\beta (V)SI+\sigma SW- (\varsigma +\iota )E,\\&\frac{dI}{dt}=\varsigma E-(\lambda +\iota )I,\\&\frac{dR}{dt}=\lambda I-(\eta +\iota )R,\\&\frac{dW}{dt}=\gamma (V)I-\xi W,\\&\frac{dT}{dt}=\frac{1}{\epsilon }[\Lambda _{T}-\tilde{\beta } VT-\mu T],\\&\frac{dT^{I}}{dt}=\frac{1}{\epsilon }[\tilde{\beta }VT-(\mu +d)T^{I}],\\&\frac{dV}{dt}=\frac{1}{\epsilon }[pT^{I}-cV].\\ \end{aligned}\quad \right. \end{aligned}$$

By getting the ideas from the optimal control researches for a multi-scale HIV model in Ref.^25^, under the influence of drug treatment, two control functions $u_1(t)$ and $u_2(t)$ are introduced into the coupled system (Eq. 16):

17 $$\begin{aligned} \left\{ \begin{aligned}&\frac{dS}{dt}=\Lambda -\beta (V)SI-\sigma SW-\iota S+\eta R,\\&\frac{dE}{dt}=\beta (V)SI+\sigma SW- (\varsigma +\iota )E,\\&\frac{dI}{dt}=\varsigma E-(\lambda +\iota )I,\\&\frac{dR}{dt}=\lambda I-(\eta +\iota )R,\\&\frac{dW}{dt}=\gamma (V)I-\xi W,\\&\frac{dT}{dt}=\frac{1}{\epsilon }[\Lambda _{T}-\tilde{\beta }(1-u_1(t)) VT-\mu T],\\&\frac{dT^{I}}{dt}=\frac{1}{\epsilon }[\tilde{\beta }(1-u_1(t))VT-(\mu +d)T^{I}],\\&\frac{dV}{dt}=\frac{1}{\epsilon }[pT^{I}-c(1+u_2(t))V],\\ \end{aligned}\quad \right. \end{aligned}$$

where the control functions $u_1(t)$ and $u_2(t)$ are bounded lebesgue integrable and represent the effects of drugs on inhibiting virus transmission and virus elimination, respectively. In addition, the coefficient $1-u_1(t)$ represents the drug effect that reduces transmission of healthy cells to infected cell as a result of interaction with the virus, while the coefficient $1+u_2(t)$ gives the another effect drug that increases the clearance of virion. The upper bounds of $u_1(t)$ and $u_2(t)$ show that the affects of the virus transmission and virus clearance. Specially, when $u_1(t)=0$ and $u_2(t)=0$, the related drugs are not inhibition virus transmission and not enhance virus removal. Naturally, each control incurs some cost, such as effective treatments usually require the existence and support of a costly public health infrastructure. Thus, we use the relative cost for the controls as the following quadratic term:

$$\begin{aligned} \frac{1}{2}[B_1u_1^2(t)+B_2u_2^2(t)], \end{aligned}$$

where $B_i$ is the cost weight for the control $u_i,i=1,2$. Next, similar to Refs.^34,35^, define the total cost objective functional for the system (Eq. 17) with the goal of minimizing free virus and infected individuals as follow:

18 $$\begin{aligned} J(u_1(t),u_2(t)):=\int _0^{t_f}\left[ A_1E(t)+A_2I(t)+\frac{B_1}{2}u_1^2(t) +\frac{B_2}{2}u_2^2(t)\right] dt, \end{aligned}$$

where $A_1$ and $A_2$ are the cost weights of exposed and infected individuals, respectively. $t_f$ is the final time. Here, our goal is to seek an optimal control $(u_1^*(t),u_2^*(t)),t\in [0,t_f]$ such that

$$\begin{aligned} J(u_1^*(t),u_2^*(t))=\textrm{min}\{J(u_1(t),u_2(t)):(u_1(t),u_2(t))\in \mathscr {G}\}, \end{aligned}$$

with $\mathscr {G}=\{(u_1(t),u_2(t)): u_i(t) \text { is lebesgue integrable}, 0\le u_i(t)\le 1,t \in [0,t_f]\}, i=1,2$ is the admissible control set. Referring to Theorem 4.1 of Ref.^35^, the following conclusion is provided.

### Theorem 5

There exists an optimal control $(u_1^*(t),u_2^*(t)),t \in [0,t_f]$ such that $J(u_1^*(t),u_2^*(t))$ subjects to the control the system (17) with nonnegative initial conditions.

### Proof

For the system (Eq. 17), define the following the Hamilton function

$$\begin{aligned} H=&A_1E(t)+A_2I(t)+\frac{B_1}{2}u_1^2(t) +\frac{B_2}{2}u_2^2(t) \\&+\lambda _1 \frac{dS}{dt}+ \lambda _2 \frac{dE}{dt}+ \lambda _3 \frac{dI}{dt}+ \lambda _4 \frac{dR}{dt}+ \lambda _5 \frac{dW}{dt}+ \lambda _6 \frac{dT}{dt}+\lambda _7 \frac{dT^{I}}{dt}+ \lambda _8 \frac{dV}{dt}, \end{aligned}$$

where $$\lambda _i,i=1,\ldots ,8$$, satisfy

$$\begin{aligned} \begin{aligned} \lambda _1'=&-\frac{\partial H}{\partial S} = (\lambda _1-\lambda _2) (\beta (V)I+\sigma W)+\lambda _1\iota ,\\ \lambda _2'=&-\frac{\partial H}{\partial E} =\lambda _2(\varsigma +\iota )-\lambda _3 \iota ,\\ \lambda _3'=&-\frac{\partial H}{\partial I} =-(\lambda _1-\lambda _2) \beta (V)S+ \lambda _3(\lambda +\iota )-\lambda _4 \lambda -\lambda _5\gamma (V),\\ \lambda _4'=&-\frac{\partial H}{\partial R} = -\lambda _1\eta +\lambda _4 (\eta +\iota ),\\ \lambda _5'=&-\frac{\partial H}{\partial W} = (\lambda _1-\lambda _2)\sigma S+\lambda _5 \xi ,\\ \lambda _6'=&-\frac{\partial H}{\partial T} = \lambda _6\frac{1}{\epsilon }[\tilde{\beta }(1-u_1(t))V+\mu ] -\lambda _7\frac{1}{\epsilon }\tilde{\beta }(1-u_1(t))V,\\ \lambda _7'=&-\frac{\partial H}{\partial T^I} = \lambda _7 \frac{1}{\epsilon }(\mu +d)-\lambda _8 \frac{1}{\epsilon } p,\\ \lambda _8'=&-\frac{\partial H}{\partial V} = \lambda _1\frac{\partial \beta (V)}{\partial V}SI- \lambda _2 \frac{\partial \beta (V)}{\partial V}SI- \lambda _5 \frac{\partial \gamma (V)}{\partial V}I + \lambda _6\frac{1}{\epsilon }\tilde{\beta } (1-u_1(t))T\\&-\lambda _7\frac{1}{\epsilon }\tilde{\beta }(1-u_1(t))T +\lambda _8\frac{1}{\epsilon }(1+u_2(t)), \end{aligned}\quad \end{aligned}$$

with transversely conditions $\lambda _i(t_f)=0,i=1,2,\ldots ,8.$ To obtain the characterization of the optimal control, it gives the following equations:

19 $$\begin{aligned} \frac{\partial H}{\partial u_i}=0, i=1,2. \end{aligned}$$

It follows from Eq. (19) that

20 $$\begin{aligned} \left\{ \begin{aligned}&B_1 u_1(t)+\lambda _6\frac{1}{\epsilon }\tilde{\beta } VT-\lambda _7\frac{1}{\epsilon }\tilde{\beta } VT=0,\\&B_2 u_2(t)+\lambda _8\frac{1}{\epsilon }cV=0.\\ \end{aligned}\quad \right. \end{aligned}$$

By solving Eq. (20), it derives

21 $$\begin{aligned} \left\{ \begin{aligned}&u_1(t)=\frac{(\lambda _7-\lambda _6)\tilde{\beta } VT}{B_1\epsilon }\triangleq \hat{u}_1(t),\\&u_2(t)=\frac{\lambda _8 cV}{B_2\epsilon } \triangleq \hat{u}_2(t).\\ \end{aligned}\quad \right. \end{aligned}$$

Combing Eq. (21) and the conditions $0\le u_i(t)\le 1,t \in [0,t_f\}, i=1,2$, the obtained optimal control is

$$\begin{aligned} \left\{ \begin{aligned}&u_1^*(t)=\max \bigg \{\min \big \{\hat{u}_1(t),1\big \},0\bigg \},t \in [0,t_f],\\&u_2^*(t)=\max \bigg \{\min \big \{\hat{u}_2(t),1\big \},0\bigg \},t \in [0,t_f].\\ \end{aligned}\quad \right. \end{aligned}$$

This completes the proof. $\square$

### Remark 4

From the Eq. (21) and the expressions of $\lambda _7', \lambda _8'$, it knows that $\hat{u}_1(t),\hat{u}_2(t)$ not only explicitly contain relevant state variables within the host such as *V*, *T* but also implicit relevant state variables between the hosts such as *S*, *I*. This means that the obtained optimal controls $\hat{u}_1^*(t),\hat{u}_2^*(t)$ may contain the relationship between the microscopic virus load per capita and the macroscopic number of infected humans, and thus, this is conducive to analyse the affect of virus load per capita on the spread of HFMD at the population level in subsequent numerical experiments.

## Numerical results

In this section, numerical simulations are given to confirm the our theoretical results and simulate the effect of virus load per capita on the spread of HFMD.

### Numerical verification of theoretical results

**Figure 1:**
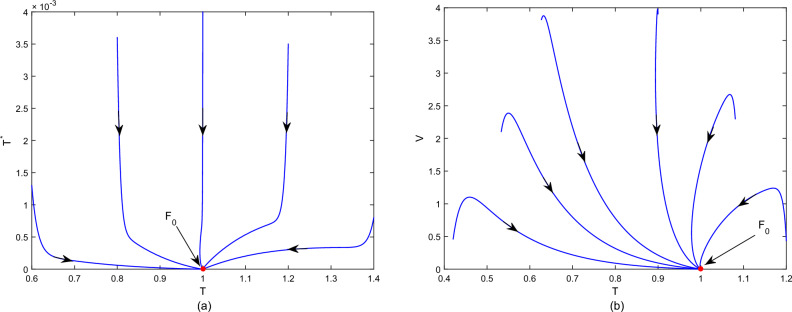
Plane phase diagram for the fast system (Eq. 3), in which $F_0$ is the disease-free equilibrium. (**a**) $T-T^*$ plane; (**b**) $T-V$ plane.

**Figure 2:**
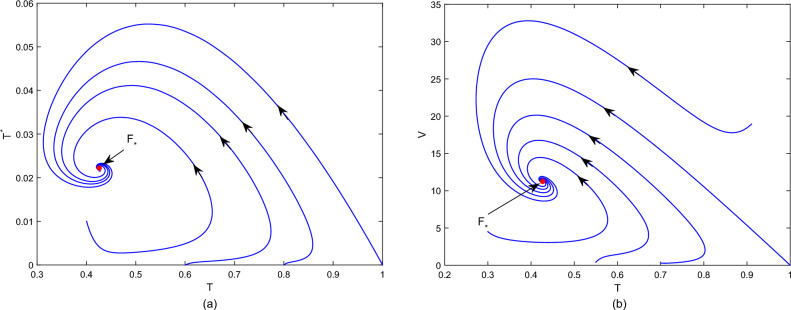
Plane phase diagram for the fast system (Eq. 3), in which $F_*$ is the positive equilibrium. (**a**) $T-T^*$ plane; (**b**) $T-V$ plane.

**Figure 3:**
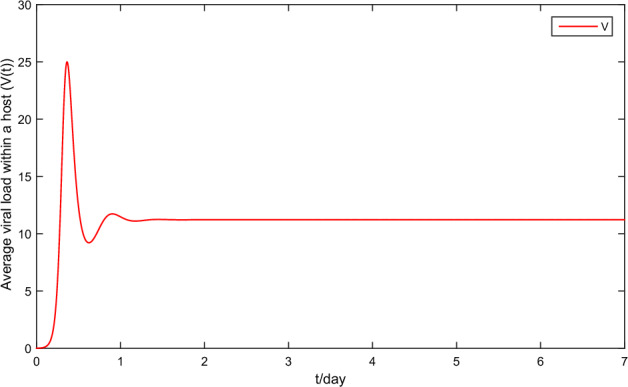
Average viral load within a host V(t) of the fast system (Eq. 3).

First, fix the parameter values in Table 2. If we choose $\tilde{\beta }=0.01$, then we obtain that the within-host system (Eq. 3) has a disease-free equilibrium $F_0=(1,0,0)$ and it calculates $R_v=0.7822$. Then, Fig. 1 shows that $F_0$ is globally asymptotically stable. If we choose $\beta =0.03$, then we obtain that the within-host system (Eq. 3) has a positive equilibrium $F_*=(0.4261,0.0222,11.2223)$ and it calculates $R_v=2.3467$. According to Theorem 1, Fig. 2 shows that $F_*$ is globally asymptotically stable. In addition, Fig. 3 also indicates that the average viral load *V*(*T*) within reach steady state rapidly, and the time required is about one day.

**Table 2:** Constant parameter values.

Parameters	Value	References
$\Lambda$	22	Assumption
$\sigma$	$4\times 10^{-6}$	^2,18^
$\iota$	$2.1\times 10^{-3}$	Assumption
$\eta$	$1.2\times 10^{-3}$	^2,18^
$\varsigma$	0.25	^2,18^
$\lambda$	0.1176	^2,18^
$\Lambda _T$	$1.72\times 10^{-5}$	^24^
$\mu$	$8.6\times 10^{-7}$	^24^
*d*	6.22	^24^
*p*	738.9	^24^
*c*	1.46	^24^
$\epsilon$	0.12	Assumption

**Figure 4:**
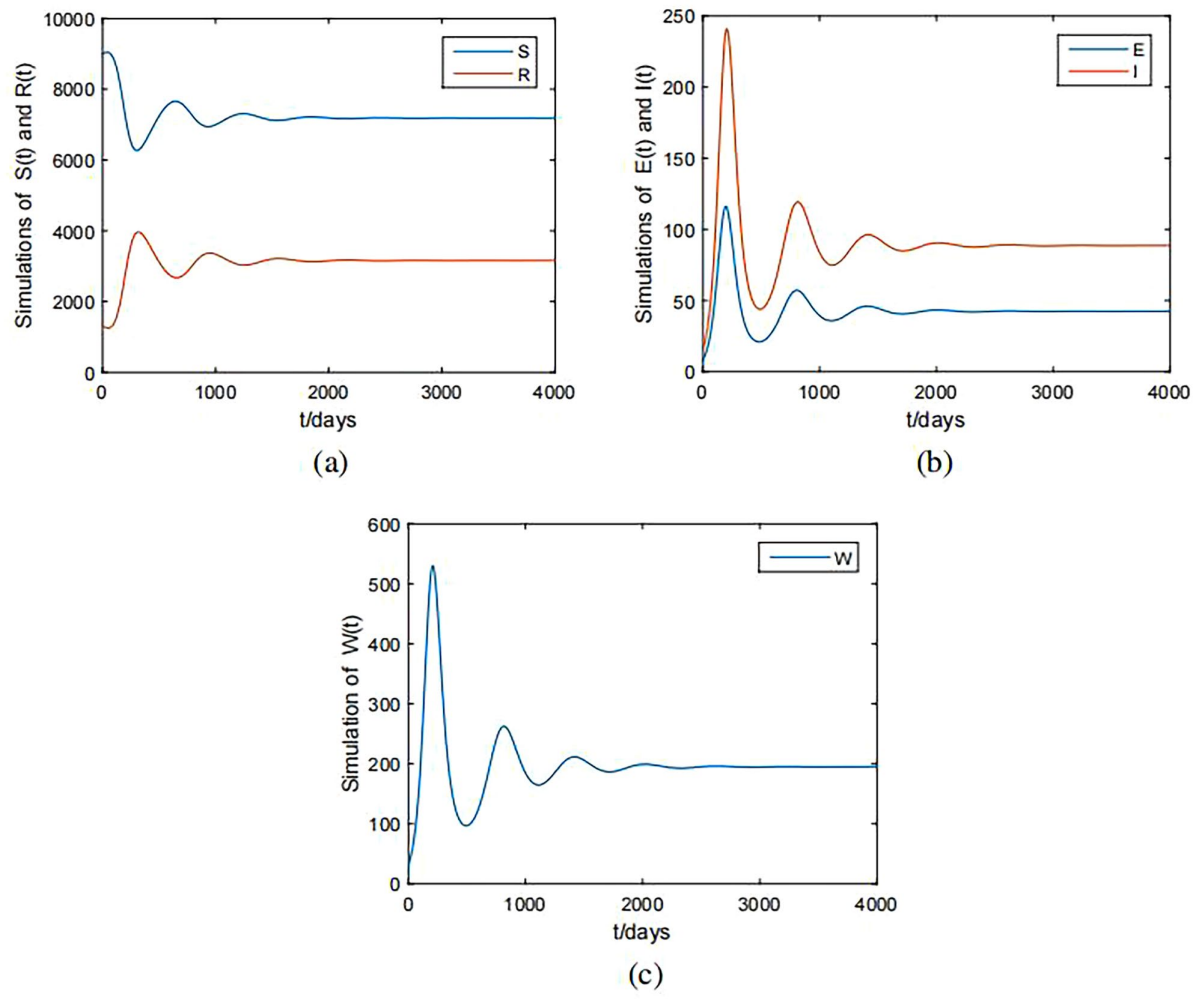
Simulations of the slow system (Eq. 1). (**a**) The state variables *S*(*t*), *R*(*t*); (**b**) The state variables *E*(*t*), *I*(*t*); (**c**) The state variables *W*(*t*).

For the system (Eq. 1), we discuss the slow system (Eq. 7). Similar to the methods in Ref.^36^, set

$$\begin{aligned} \beta (V)=\frac{r_WV_*}{K_W},\gamma (V_*)=\frac{\tilde{r}_WV_*}{K_W}, \end{aligned}$$

where the parameters $r_W$ and $\tilde{r}_W$ are virus transmission rate and virus shedding rate, respectively. $K_W$ is the threshold of the viral load in a host may need to cross in order to transmit the infection. If we take $r_W=3.5\times 10^{7} ,\tilde{r}_W=0.46, K_W=5$ and use the $V_*$ from Fig. 2, then by simple calculation, it follows that $R_h=1.4581$. Figure 4 shows that the system (Eq. 7) is uniformly persistent, which means HFMD is persistent.

### The effect of virus load per capita on HFMD transmission

**Figure 5:**
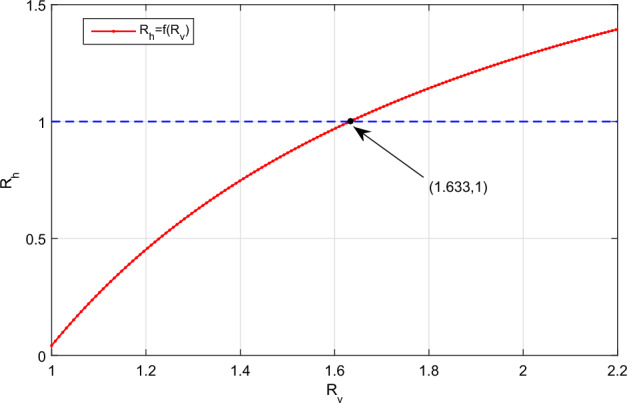
The relationship between the two basic reproduction numbers $R_h,R_v$ (take $R_h=f(R_v)$).

**Figure 6:**
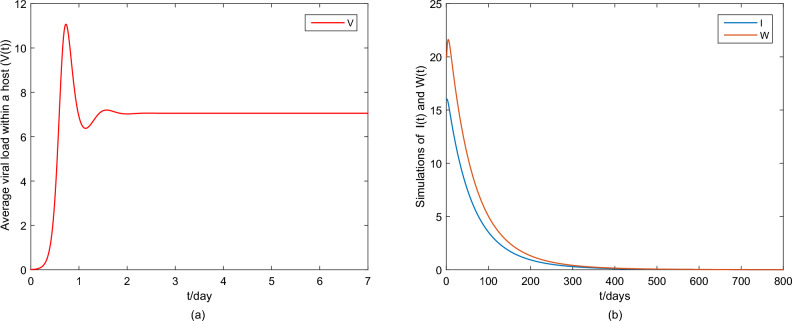
(**a**) Simulation the state variable *V*(*t*) of the fast system (1); (**b**) Simulation the state variables *I*(*t*), *W*(*t*) of the slow system (3).

The affect of virus load per capita on the spread of HFMD will be discussed. That is, assuming that the medical authorities can quickly reduce the virus load per capita to a lower level, even if the virus cannot be eliminated immediately, we analyse the impact of this measure on the HFMD transmission. In addition, note that the basic reproduction number is the key threshold for evaluating the spread of the epidemic. It is clear that $R_h$ decreases as $R_v$ decreases by comparing the expressions of $R_h$ and $R_v$. If we continuously change $\tilde{\beta }$ for 0.013 to 0.03 and fix the other parameter values same as in Figs. 2 and 4, then the relationship between the two basic reproduction numbers $R_h$ and $R_v$ is indicated in Fig. 5. Figure 5 illustrates that when $1<R_v<1.633$, we have $R_h<1$. This implies that during the infection period, as long as the per capita viral load is controlled at a low level even if it cannot be eliminated immediately, the disease will become extinct. In addition, if we choose $\tilde{\beta }=0.02$ ($R_h=0.9167,R_v=1.5644$), then Fig. 6a shows the state variable *I*(*t*) of the slow system (Eq. 1) tends to 0, meanwhile the state variable *V*(*t*) of the fast system (Eq. 3) tends to be a constant greater than 0. From the above discussion, it comes to a very important conclusion: although there are no targeted drugs for HFMD, we can actively treat HFMD and control the viral load per capita to a lower value, which can also eliminate the disease.

## Optimal control of the actual epidemic application

This section numerically discuss the influences of optimal control of drug therapy in human body on HFMD transmission at the between-host level, by using relevant HFMD data of Zhejiang Province, China. Only children under 5 years old are considered, as they are the group with the highest risk of the disease^1–3^. Note that HFMD transmission is influenced by climate and population concentration^12,13,15,19^. In addition, our between-host model is an autonomous differential equation with constant coefficients. The numerical experiment needs to avoid the influence of periodic (climate factors) or sudden (change in children’s concentration) objective factors. Therefore, in order to avoid above objective factors, we choose the period from April 1, 2021 to June 30, 2021 when climatic conditions and children’s aggregation have little change. Furthermore, it should be noted that the period from onset to recovery of HFMD is extremely short, which is generally within 10 days. Therefore, the time period (April 1 to June 30) contains multiple popular waves, which is enough for us to observe the fitting effect of our model.

### Data fitting

The daily newly reported HFMD cases are obtianed from Health Commission of Zhejiang Province (HCZP)^37^, and temographic information is acquired from Zhejiang Provincial Bureau of Statistics (ZPBS)^38^. According to the experimental data of HFMD virus dynamics model in Ref.^26^, the units of $V(t),T^I(t)$ and *V*(*t*) are $cells/10^{-5} ml, cells/10^{-5} ml$ and $virus-numbers/10^{-5} ml$, respectively. According to Refs.^26,37,38^ and getting $W(0)=120$ from Refs.^14,15^ we set the initial conditions of the system (Eq. 16) and the system (Eq. 17) as

22 $$\begin{aligned} (S(0),E(0),I(0),R(0),W(0),T(0),T^I(0),v(0))=&(2.95\times 10^6,125,95,96,120, 10,0.02,0). \end{aligned}$$

Some parameters related to HFMD, such as the recovery rate $\lambda$, the natural cell mortality $\mu$ and so on, are from the previous research papers, some of which can also be seen in Table 2. Similar to the approach in Ref.^39^, combining the related research results in Refs.^32,33^, we can reasonably assume that

$$\begin{aligned} \beta (V)=k_\beta V,\gamma (V)=k_\gamma V, \end{aligned}$$

where $k_\beta$ and $k_\gamma$ need to be estimated. In addition, all the unknown parameters $k_\beta , k_\gamma , \sigma$ are estimated by using our proposed algorithm based on BP neural network in Refs.^20,21^. The parameter estimation process based on BP neural network is described as:

1. BP neural network is designed as a three-layer structure of input layer, middle layer and output layer. The input vector is the daily reported HFMD cases in Zhejiang Province, China, from April 1, 2021 to June 30, 2021. The output vector consists of $\sigma ,k_\beta ,k_\gamma$.
2. Train data: The numbers of neurons in the input layer, the middle layer and the output element are 91, 200 and 3, respectively. Then, based on Latin hypercube sampling technology, model (Eq. 16) is used to generate 2000 groups of training data for training.
3. Estimate parameters: Substituting the daily reported HFMD cases in Zhejiang Province, China, from April 1, 2021 to June 30, 2021 the trained BP neural network to evaluate the unknown parameters $\sigma ,k_\beta ,k_\gamma$. Then, all the parameter values are listed in Table 3. It should noted that some parameter values Table 3 are different from that in Table 2. Tables 2 and 3 have different roles in our paper. Table 2 is mainly to show the correctness of theoretical results related to system stability. Therefore, some parameter values are artificially set or derived from previous studies, such as $\Lambda , \sigma ,k_\beta ,k_\gamma$ and so on. However, Table 3 is a fitting of actual epidemic data from specific areas of primary sources, which is intended to demonstrate the effectiveness of optimal control. Therefore, some parameters in the two tables are quite different, which is normal.

**Table 3:** Constant parameter values.

Parameters	Value (per day)	References
$\Lambda$	1859	^38^
$\iota$	$7.12\times 10^{-4}$	^38^
$\eta$	$1.2\times 10^{-3}$	^2,18^
$\varsigma$	0.25	^2,18^
$\lambda$	0.1176	^2,18^
$\Lambda _T$	$1.72\times 10^{-5}$	^24^
$\mu$	$8.6\times 10^{-7}$	^24^
*d*	6.22	^24^
*p*	738.9	^24^
*c*	1.46	^24^
$\epsilon$	0.12	Assumption
$\sigma$	$4\times 10^{-6}$	Estimation
$k_\beta$	$1.83\times 10^{-8}$	Estimation
$k_\gamma$	$3.57\times 10^{-9}$	Estimation

**Figure 7:**
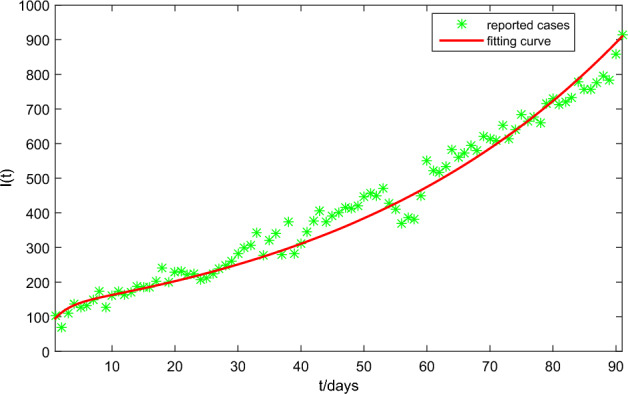
The system (Eq. 16) fits the reported HFMD data in Zhejiang Province, China, from April 1, 2021 to June 30, 2021.

Based on the parameter values in Table 3 and the initial conditions (Eq. 22), Figure 7 shows the numerical simulation of the system (Eq. 16) on the number of HFMD cases in Zhejiang Province, China, from April 1, 2021 to June 30, 2021. Figure 7 also indicates that the number of new HFMD cases has been on the rise during this period. HFMD is a self-limiting disease that can be cured without medication. Therefore, many infected people fail to seek medical treatment in time or do not seek medical treatment. In the next subsection, we will use the fitting curve in Figure 1 to analyse the impact of drug treatment prevention and control on the transmission of HFMD.

#### Remark 5

The detailed process of estimating unknown parameters by the algorithm based on BP neural network is cumbersome, and can be seen in Refs.^20,21^. Therefore, it is omitted here.

### Simulation optimization control

This subsection analyses the control measures for timely drug treatment in a short period of time. To this purpose, combining the model (Eq. 17) with the fitting curve of Figure 7 is discussed, i.e., the influences of the optimal controls in within-host of the system (Eq. 17) are on the infected number *I*(*t*) in between-host part of the system (Eq. 17). To facilitate analysis, the following assumptions are needed:

$(\textbf{H1})$ During the implementation of optimal control, every infected patient can receive timely treatment. This can be ensured through the joint efforts of public health authorities, child-related authorities and guardians of children.

$(\textbf{H2})$ Under the corresponding treatment costs, the average viral load of an infected patient can be controlled to the optimal control solution obtained by the system (17).

**Figure 8:**
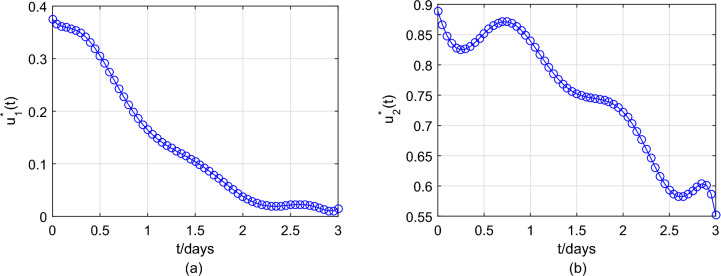
The solved optimal controls for system (Eq. 17).

**Figure 9:**
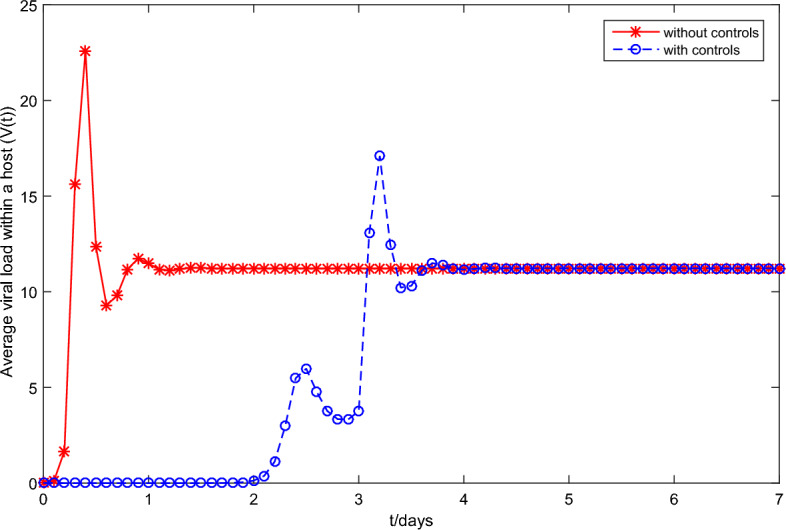
Dynamics of *V*(*t*) of system (Eq. 17) with and without optimal controls.

**Figure 10:**
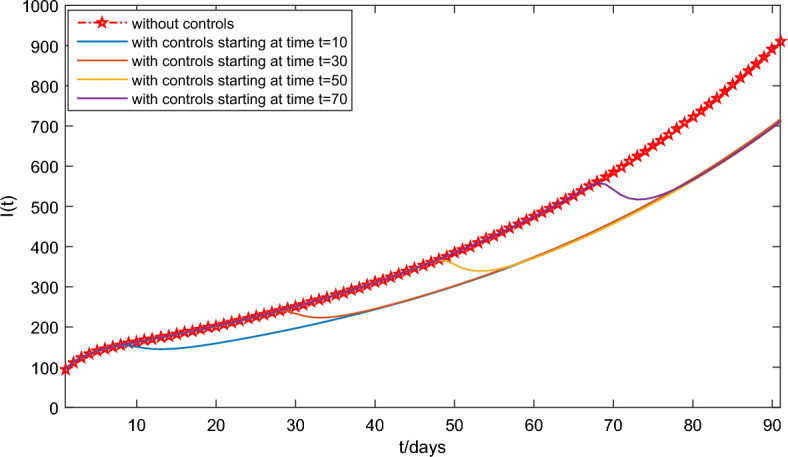
Dynamics of *I*(*t*) of the system (Eq. 17) with and without optimal controls, with control time starting at $t=10,30,50,70$, respectively.

Under the conditions ($\textbf{H1}$) and ($\textbf{H2}$), it will solve the optimal problem in **optimal control** section. Based on the researches on HFMD in Chinese mainland has been conducted in recent years^32,33^, the economic loss caused by latent individuals and infection to society is about 10 yuan, or 20 yuan, respectively, and the cost of positive treatment for HFMD infection is about 200 yuan. Thus, set $A_1=10, A_2=20,B_1=200/2=100,B_2=200/2=100$. It should be noted that, the optimal control method adopted is the terminal time optimization control in the optimal control of infectious disease dynamic system. The biological meaning is that the terminal time t must be less than the average recovery time of the infected person $1/\varsigma =4$. Therefore, we set $t_f=3$ days for object function (Eq. 18). In the previous researches on the fast-slow system of infectious diseases in Refs.^24,27,39^, the value of $\epsilon$ have usually been taken the ratio of the average duration of an infected person’s viral load in an unstable state to the average duration of the infection period, because the infected person is constant when the viral load is in a stable state. According to Ref.^26^, the average duration of an infected HFMD individual’s viral load in an unstable state is slightly less than 1 day and the average duration of the infection HFMD period is about 8.5 days. Therefore, we let $\epsilon =\frac{1}{8.5}=0.12$. Take the parameter values in Table 3 and the initial conditions (Eq. 22) for the system (Eq. 17). Note that $u_1(t)$ and $u_2(t)$ affect the ability of infected cells to spread with respect to the rate $\tilde{\beta }$ and the intensity of virus clearance with respect to the rate *c*, respectively. Thus, in order to analyze the influence of optimal control on the ability of infected cells, the intensity of virus clearance, and the whole disease transmission, we only need to discuss that the influence of $u_1(t)$ and $u_2(t)$ on total cost objective function (Eq. 18). Figure 8 shows that the optimal $u_1^*(t),u_2^*(t)$ are a decreasing trend, meanwhile $u_1^*(3)=0.0149,u_2^*(t)=0.5518$, which means the effects of optimal controls with respect to virus load per capita are first strong and then weak (seeing Fig. 9). Figure 9 indicates that the virus load per capita *V*(*t*) of the system (Eq. 17) with optimal controls is lower than that without optimal controls. The lower virus load in an infected individual implies the less transmission capacity, which is illustrated in Fig. 10. Moreover, Fig. 10 indicates the comparison between the of infected number *I*(*t*) under optimal controls with respect to the system (Eq. 16) at different implemented times $t=10, 30, 50,70$ and that without optimal controls with respect to the system (Eq. 17), meanwhile also reflects two phenomena:

1. The number of newly infected individuals under optimal control is lower than those without optimal control.
2. When the number of newly infected individuals continues to rise, the earlier the control measure is implemented, the smaller the total number of infected persons, that is, the earlier the control measure is implemented, the more conducive it is to reduce the spread of the disease.

In summary, the drug control strategies reducing the virus load per capita can effectively prevent large-scale outbreaks of HFMD.

## Discussion

In this paper, a multi-scale HFMD model was proposed. It notes that the previous studies were independent analysis of between-host and within-host dynamics for the spread of HFMD. We constructed the relation between between-host system and within-host system by establishing the transmission rate of human-human $\beta$ and the rate of virus shedding from the host to the external environment $\gamma$ as a function of the viral load in a host *V*(*t*), which brings new challenge problems. Due to the dynamical processes of the within-host and between-host develop at different time scale, by using the fast-slow analysis method, some stability analyses were provided for the proposed model. Through define two basic reproduction number $R_v$ (for fast system) and $R_h$ (for slow system), we investigated the global stabilities of positive equilibrium and boundary equilibrium for fast system, and the global stability of boundary equilibrium and uniform persistence for the slow system.

When we discussed the affect of virus load per capita on the spread of HFMD, we have found an interesting result: during the infection period, as long as the per capita viral load is controlled at a low level even if it cannot be eliminated immediately, the disease will become extinct (see the analysis of Fig. 5), which provides a favorable basis for the prevention and control of HFMD. In addition, the optimal control on the within-host part of the purposed multi-scale HFMD system was formulated, and its related theories have been rigorously proven. Furthermore, the optimal control measure was applied to the actual HFMD epidemic analysis in Zhejiang Province, China, and thus, an important conclusion was obtained: when the number of newly infected individuals continues to rise, the earlier implementation of optimal control would help reduce the spread of disease.

## Data Availability

The datasets analysed during the current study are not publicly available due to that the data from real-time reporting on infectious diseases can only be used for scientific research and must not be reproduced, but are available from the corresponding author on reasonable request.
